# Comparative Study of the Antioxidant Mediated Antiaging Properties of Endocarp Extracts From *Phoenix dactylifera* and *Phoenix dactylifera L*. via In Silico and In Vitro Analysis

**DOI:** 10.1002/fsn3.72388

**Published:** 2026-09-21

**Authors:** Farheen Asaad, Mazhar Abbas, Waqas Haider, Naveed Munir, Maha Gul Zafar, Muhammad Riaz, Muhammad Arshad, Muhammad Arfan Zaman, Tariq Hussain, Muhammad Adnan Ayub, Hasan Ejaz, Quzi Sharmin Akter

**Affiliations:** ^1^ Department of Basic Sciences (Section Biochemistry) University of Veterinary and Animal Sciences (Jhang Campus) Jhang Pakistan; ^2^ Department of Biomedical Lab Sciences, School of Health Sciences University of Management and Technology Lahore Pakistan; ^3^ Department of Allied Health Sciences University of Sargodha Sargodha Pakistan; ^4^ Department of Pathobiology (Section Parasitology) University of Veterinary and Animal Sciences (Jhang Campus) Jhang Pakistan; ^5^ Department of Basic Sciences (Section Pharmacology) University of Veterinary and Animal Sciences (Jhang Campus) Jhang Pakistan; ^6^ Department of Chemistry University of Sahiwal Sahiwal Pakistan; ^7^ Department of Clinical Laboratory Sciences, College of Applied Medical Sciences Jouf University Sakaka Saudi Arabia; ^8^ Department of Genetics and Animal Breeding, Faculty of Animal Science and Veterinary Medicine Patuakhali Science and Technology University Patuakhali Bangladesh

**Keywords:** anti‐aging, ECM‐degrading, *Phoenix dactylifera*, *Phoenix dactylifera* *L.*

## Abstract

The rising demand for sustainable cosmeceuticals has driven us to explore the botanical source for anti‐aging application. This comparative study explores the antioxidant mediated anti‐aging potential of two varieties of dates *Phoenix dactylifera* (Zahidi dates) and *Phoenix dactylifera L.* (Piarom dates), through both in vitro and in silico analysis. It aims to evaluate antioxidant potential, inhibition of anti‐aging protein and binding interaction of phytochemicals against the target of anti‐aging proteins. To achieve this, total phenolic and flavonoid content was analyzed, and radical scavenging activity was determined by DPPH analysis. The antioxidant enzymes SOD, catalase and peroxidase activity were assessed. Results revealed higher phenolic (92 ± 1.15 mg GAE/g), flavonoid (39.2 ± 0.11 mg CE/g) and % inhibition of DPPH (81.2 ± 1.46) in *Phoenix dactylifera* as compared to *Phoenix dactylifera L.,* revealing its high antioxidant potential. *Phoenix dactylifera* exhibited a greater anti‐aging potential against ECM‐degrading proteins than *Phoenix dactylifera L*., as evidenced by the inhibition percentages of elastase (49.69% ± 1.15%), hyaluronidase (51.65% ± 1.49%) and collagenase (58.41% ± 1.98%). LCMS analysis identified key phytochemicals that are madecassoside, glutaric acid, benzoquinoline, myrtenol and pimelic acid. FTIR and UV spectroscopy confirmed the presence of hydroxyl and phenolic compounds. These in vitro analyses were further validated by in silico pharmacokinetics that suggest the majority of phytochemicals have high skin permeability, high GI absorption and suitable bioavailability scores, with phosphatidylcholine (*Phoenix dactylifera L*.) having a LogKp value of −0.27, and furostane ( *Phoenix dactylifera* ) having a LogKp value of −3.16. The molecular docking analysis revealed 6‐sec‐Butylquinoline ( *Phoenix dactylifera* ) and isonipecotic acid (*Phoenix dactylifera L.)* outperforming with inhibition values reaching up to −11.8062 and −15.82 kcal/mol against ECM‐degrading protein targets. These findings support *Phoenix dactylifera* 's antioxidant mediated‐anti‐aging properties in comparison to *Phoenix dactylifera L*., prompting subsequent in vivo and clinical research to confirm its effectiveness.

## Graphical abstract

This comparative study demonstrates that *Phoenix dactylifera* possesses higher anti‐aging potential compared to *Phoenix dactylifera* L., which may be attributed to their higher phenolic and flavonoid contents along with stronger free radical scavenging capacity. Both extracts exhibited significant inhibitory activity against extracellular matrix (ECM)‐degrading enzymes, including elastase, tyrosinase, and hyaluronidase, indicating their potential in mitigating skin aging processes. In silico results also confirmed skin permeability and gastrointestinal absorption profiles of phytochemicals, supporting their suitability for dermal applications.

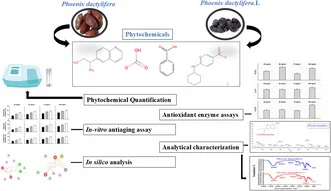

## 1 Introduction

The skin, comprising the epidermis and dermis, serves as a protective barrier. The dermal extracellular matrix (ECM) provides structural support and regulates fibroblast function (Lee et al. 2021). Aging results from both intrinsic and extrinsic factors such as DNA damage, mitochondrial dysfunction, and oxidative stress. Free radicals and oxidative stress cause structural and chemical compositional change in skin by oxidizing lipids and proteins. These ROS cause cell membrane damage and DNA damage, eventually leading to cell death (Sadiq 2023). Free radicals also may cause the degradation of hyaluronic acid (activation of hyaluronidases), which is responsible for the proper hydration of the skin and its firmness (Chaudhary et al. 2023). Photodamage is the primary extrinsic factor that contributes to skin aging, which in turn disrupts ECM balance and activates the ECM‐degrading proteins elastase, collagenase, tyrosinase, and hyaluronidase. It results in degrading skin elasticity, moisture content, and firmness. It also triggers chronic inflammation via SASP (senescence‐associated secretory phenotype), driving skin aging by the appearance of wrinkles and leathery skin (Zhang et al. 2024).

The topical use of botanical extract has revolutionized the skincare industry due to its antioxidant potentials (Merecz‐Sadowska et al. 2021). Plant‐derived raw materials offer several beneficial properties, including moisturizing, barrier repair, antioxidant, photoprotective, and anti‐inflammatory effects that contribute to anti‐aging activity through multiple biological pathways (Nowak‐Perlak et al. 2025). The moisturizing plant ingredients, particularly polysaccharides and glycosides rich in hydroxyl groups, can retain water molecules through hydrogen bonding. Plant species like licorice and aloe polysaccharides improve external hydration and enhance aquaporin expression in epidermal keratinocytes (Michalak 2022). Plant materials that support barrier repair are abundant in essential fatty acids that help strengthen the skin barrier and maintain overall skin health. Antioxidant‐rich botanical products mainly consist of phenolic compounds, carotenoids, and vitamins, which help in neutralizing free radicals and reactive oxygen species (ROS), thereby preserving the redox balance within skin cells (Xie et al. 2024). Certain plant phytocompounds also provide sunscreen and skin‐whitening benefits. Components such as flavonoids, phenolic quinones, phenylpropanoids, and carotenoids possess conjugated structures capable of absorbing ultraviolet radiation, allowing them to function as natural photoprotective agents (Tanveer et al. 2023). In addition, salidroside from *Rhodiola rosea* suppresses melanin production and tyrosinase activity. Plant phytoconstituents such as grape seed oil can reduce inflammation and relieve chemically induced contact dermal issues (Costa et al. 2022).

Phenolic compounds, including caffeic, ferulic, gallic, and sinapic acids, possess strong antioxidant and anti‐inflammatory activities that contribute to photoprotection and anti‐photoaging effects (Serra et al. 2023). These compounds reduce oxidative stress, suppress ROS generation, inhibit matrix metalloproteinases (MMPs), and protect collagen and elastin from UV‐induced degradation. Caffeic acid and its derivatives inhibit UV‐induced MMP‐1 expression and inflammatory signaling pathways, while ferulic acid enhances the stability and efficacy of vitamins C and E, protecting skin cells from oxidative damage and improving skin hydration and texture (del Carmen Villegas‐Aguilar et al. 2023). Sinapic acid also reduces ROS production and supports collagen synthesis by suppressing MAPK/NF‐κB signaling pathways (Islam et al. 2025). Tannins and proanthocyanidins derived from sources such as grapes, tea, cranberry, pine bark and peanut skin also demonstrate significant antioxidant and anti‐inflammatory properties (Javed et al. 2025). These compounds reduce ROS production, suppress inflammatory mediators such as COX‐2 and iNOS inhibit collagenase and elastase activity and decrease MMP expression, thereby preventing collagen degradation and UV‐induced photoaging (Yusuf et al. 2024). Isoflavones such as genistein from soybeans inhibit UV‐induced inflammation, decrease MMP‐1 expression and stimulate collagen synthesis. Catechins from *Camellia sinensis* , particularly EGCG, effectively scavenge ROS, inhibit collagenase and elastase activity, suppress MMP production, and reduce pigmentation by inhibiting tyrosinase activity (Păcularu‐Burada et al. 2024). Quercetin, myricetin, fisetin, baicalin, hesperidin and naringenin protect against UV‐induced oxidative stress, inflammation, collagen degradation and wrinkle formation by regulating signaling pathways such as MAPK, ERK and NF‐κB while enhancing antioxidant defense systems (Niu et al. 2022). Dates and date palms have botanical importance due to their adaptability to arid environments, nutritional value, economic significance and cultural and culinary importance (Alkhoori et al. 2022). *Phoenix dactylifera* (Zahidi dates), like other date varieties, are a good source of energy and contain essential nutrients such as vitamins, minerals, carotenoids, phenolics, flavonoids, antioxidants and dietary fiber (Ali and Mohammed 2021). They are particularly rich in potassium, magnesium and iron (Hussain et al. 2020). *Phoenix dactylifera L*. (Piarom dates) are the best source of energy and contain essential nutrients such as vitamins (particularly B vitamins), minerals (like potassium, magnesium, and iron) and dietary fiber (Alam et al. 2023). Numerous antioxidants, including flavonoids like quercetin, kaempferol and catechin, are found in dates. Dates contain carotenoids that have antioxidant properties, such as lutein and beta‐carotene that contribute to alleviating oxidative stress and inflammation (Mauludiyana et al. 2023). In addition to their great nutritional value, date fruits have been used in ethnobotanical traditions to treat a variety of ailments, including liver infections, headaches, respiratory problems, diarrhea and constipation. The antioxidant, anti‐inflammatory, antimutagenic, anticancer, antiviral, antifungal, gastroprotective, hepatoprotective, nephroprotective, neuropharmacological and analgesic properties of date fruits have been demonstrated to be substantial (Alkhalaf et al. 2023; Kharal et al. 2024; Younas et al. 2020).

This comparative investigation assessed the anti‐aging potential of *Phoenix dactylifera* (Zahidi dates) and *Phoenix dactylifera L*. (Piarom dates) using integrated in vitro and in silico analysis. Antioxidant potential was determined through the estimation of total phenolic content (TPC), total flavonoid content (TFC), and DPPH radical scavenging activity. The radical scavenging potential was further validated by enzymatic antioxidant assays evaluating superoxide dismutase (SOD), catalase, and peroxidase activities. The anti‐aging efficacy of the extracts was examined by assessing their inhibitory effects against extracellular matrix (ECM)‐degrading enzymes, including elastase, tyrosinase, and hyaluronidase. Phytochemical constituents were identified using LC–MS analysis and subsequently characterized by FTIR and UV–visible spectroscopy. Additionally, the experimental findings were complemented by in silico pharmacokinetic evaluation and molecular docking studies to investigate the binding interactions and affinities of the identified compounds with the target enzymes elastase, tyrosinase, and hyaluronidase.

## 2 Materials and Methods

### 2.1 Chemicals and Reagents

All the reagents and chemicals used in this study were of analytical grade. For extraction of bioactive compounds, organic solvents were purchased from Merck (Darmstadt, Germany). For in vitro antioxidants, catechin, gallic acid, Folin–Ciacolteu (FC) reagent and synthetic oxidant scavenger 2,2‐diphenyl‐1‐picrylhydrazyl radical (DPPH) were purchased from Sigma Aldrich USA. Different salts like sodium hydroxide (NaOH), aluminum chloride (AlCl_3_), sodium nitrate (Na_2_NO_3_) and sodium carbonate (Na_2_CO_3_) used in this study were also purchased from Merck. Tyrosinase, L‐DOPA, Elastase, Tris Buffer, Succinyl‐trialanine 4‐nitranilide, Hyaluronidase, Phosphate Buffer, Hyaluronic Acid, and Acidic Albumin Acid used for the antiaging assay were purchased from Sigma and Merck.

### 2.2 Collection of Endocarp and Extraction

Dates were purchased from local market of Jhang Sadar, Punjab, Pakistan during September 2024 and authenticated by Botany Lecturer “Zayira Rafique,” at CVAS Jhang. The local names of the cultivars used in this study are (Zahidi dates) and (Piarom dates) for *Phoenix dactylifera* and *Phoenix dactylifera L*, respectively. Then dates were washed with tap water and dried at room temperature (35°C). These were then cut with a sharp knife and the endocarp was collected after removing the seeds. The upper transparent layer (endocarp) was carefully separated and stored in a jar. Subsequently, 100 mg of endocarp was dissolved in 100 mL of distilled water at powder‐to‐solvent ratio of 1:1 and shaken for approximately 1 h at 40°C. The extract was then filtered and lyophilized at −50°C. Finally, the lyophilized product was kept at room temperature for further analysis (Galefi et al. 2023).

## 3 Phytochemical Quantification

### 3.1 Total Phenolic Contents (TPC)

The total phenolic contents of the extracts was analyzed through the method reported by Aryal et al. (2019). For this assay, 300 μL of 25–100 mg/mL of both extracts of *Phoenix dactylifera* and *Phoenix dactylifera L*. were mixed with 500 μL of Folin–Ciocalteu's reagent. After 4–5 min of incubation, 0.8 mL of 7.5% sodium carbonate was added. The mixture was kept in the dark at room temperature for 30 min. Absorbance was measured at 765 nm. Results were expressed as mg Gallic acid equivalents (GAE).

### 3.2 Total Flavonoid Contents (TFC)

The complete flavonoid concentration was examined as per the methodology described by Nugroho et al. (2023). For this analysis, 400 μL of 5% sodium nitrate solution was added to 1 mL of test sample of *Phoenix dactylifera* and *Phoenix dactylifera L*. endocarp extract (25–100 mg/mL). The sample was left for 15 min at room temperature 35°C. Following the addition of 700 μL of 10% aluminum chloride to each test tube, 1 mL of 1 M sodium hydroxide solution was added. A spectrophotometric cuvette was filled with 500‐μL test sample, and the absorbance at 510 nm was measured to determine the TFC as mgCE.

## 4 Antioxidant Assay

### 4.1 Radical Scavenging Activity by DPPH Assay

DPPH (2,2‐Diphenyl‐1‐picrylhydrazyl) is used to determine the antioxidant activity reported by Zafar et al. (2025). Stock solutions of 25, 50, 75, and 100 mg/mL of endocarp extract of *Phoenix dactylifera* and *Phoenix dactylifera L*. were prepared for radical scavenging activity. From the stock solution, 50 μL was poured into a sterilized test tube along with the addition of 5 mL of 0.004% methanol solution of DPPH. The entire process was carried out in a dark environment to prevent photoreaction. All test tubes were left at room temperature for a normal reaction for 30 min. After the normal reaction, 500 μL of the sample solution was transferred into a spectrophotometric cuvette, and absorbance was measured at 517 nm.

$$\text{DPPH scavenging activity}\left(\%\right)=\left[\frac{\left({\mathrm{Abs}}_{\text{control}}-{\mathrm{Abs}}_{\text{sample}}\right)}{{\mathrm{Abs}}_{\text{control}}}\right]\times 100$$

Control or blank contains all reagents except the test compound, while the sample was the test compound. Antioxidant activity taken as percentage inhibition and IC_50_.

## 5 Antioxidant Enzyme Assays

### 5.1 Superoxide Dismutase (SOD) Activity

The suppression of the enzyme's ability to reduce nitroblue tetrazolium (NBT) photochemically was used to calculate Superoxide Dismutase activity. To initiate the reaction, 3 mL of reaction mixture (13.33 mM methionine, 75 μM NBT, 0.1 mM EDTA, 50 mM phosphate buffer [pH 7.8], 50 mM sodium carbonate, and 0.1 mL enzyme extract) was mixed with about 0.1 mL of 2 mM riboflavin. In comparison to tubes without enzyme, the absorbance reading was lowered to 50% when the absorbance was measured at 560 nm, and one unit of enzyme activity was calculated as that amount of enzyme (Edo et al. 2025).

### 5.2 Catalase (CAT) Activity

The catalase activity of *Phoenix dactylifera* and *Phoenix dactylifera L*. was determined following the standard protocol (Raju and Rao 2021). By measuring the amount of H_2_O_2_ that disappeared, catalase activity was measured. About 50 μL of diluted enzyme extract and 1.5 mL of 0.1 mM phosphate buffer (pH 7) were added to 3 mL of reaction mixture, along with 0.5 mL of 75 mM H_2_O_2_. After observing a one‐minute drop in absorbance at 240 nm, the amount of H_2_O_2_ decomposed was used to calculate the enzyme activity.

### 5.3 Peroxidase (POD) Activity

The peroxidase activity of the test sample was measured by following the method of Kohatsu et al. (2021). The reaction mixture included o‐dianisidine (0.25 mM), hydrogen peroxide (1 mM), 0.1 M sodium phosphate buffer pH 6.0, and an enzyme aliquot. The oxidation of o‐dianisidine in the presence of hydrogen peroxide caused a change in absorbance of 0.02–0.07 per minute at 460 nm. The quantity of enzyme required to oxidize 1 μmol o‐dianisidine/min is known as one unit of enzyme activity (ε460 = 11.3 mM^−1^ cm^−1^). For additional research, the leaf homogenate with the highest peroxidase activity was used.

## 6 In Vitro Antiaging Assay by Different Enzymatic Method

### 6.1 Tyrosinase Inhibition Assay

The in vitro inhibition of tyrosinase was measured using the method of Srisuksomwong et al. (2023) with slight modification. L‐DOPA (100 μg/mL) and mushroom tyrosinase (25 U/mL) were used as the substrate and enzyme, respectively. The assay was carried out in a 96‐well microplate by mixing 10 μL of the ethanol‐solubilized sample at different concentrations with 20 μL of tyrosinase solution and 150 μL of 100 mM sodium phosphate buffer (pH 6.8). The mixture was incubated at 37°C for 10 min. Subsequently, 20 μL of L‐DOPA was added and the absorbance was recorded at 475 nm. Control containing kojic acid (1 mg/mL) as a positive control to compare the inhibitory potential.

### 6.2 Elastase Inhibition Assay

The inhibition of enzyme elastase was measured by following the modified protocol of Younis et al. (2022). Stock solutions with concentrations of 25, 50, 75, and 100 mg/mL were made. The substrate was N‐succinyl‐Ala‐Ala‐Pro‐Phe p‐nitroanilide (2 mM), while the enzyme source was porcine elastase (1.4 IU). First, the sample extract (25–100 mg/mL) from the stock solution was mixed with porcine elastase in 0.1 M Tris buffer solution to reach 0.1 mL, and the mixture was incubated for 5 min. Next, 25 μL of Tris–HCl buffer containing 1 mM N‐succinyl‐(Ala)_3_‐p‐nitroanilide was added. After that, the well plate was incubated at room temperature for 50 min. A microplate reader was used to measure the absorbance variation at 420 nm.

### 6.3 Hyaluronidase Inhibition Assay

The hyaluronidase activity was analyzed by following the method of Prashanth et al. (2025) with slight modification. In this assay, 300 IU/mL of bovine hyaluronidase solution was prepared in 0.1 M acetate buffer and incubated for 20 min at 37°C. A concentration of plant extract (25–100 mg/mL) was added to a 5% DMSO solution. After that, the reaction mixture was incubated for 20 min with 100 μL of 12.5 mM CaCl_2_ added. Sodium hyaluronate (1.2 mg/mL) was then added in 250 μL and left for 40 min. Following treatment with 100 μL of each 0.4 M NaOH and 0.4 M potassium borate, the reaction mixture was heated for 3 min. Then, 3 μL of p‐dimethylaminobenzaldehyde solution was added after cooling, and the mixture was incubated for 20 min at 37°C. The absorbance was recorded at 585 nm.

## 7 Analytical Characterization of Extract

### 7.1 Liquid Chromatography Mass Spectrometry (LC–MS)

The *Phoenix dactylifera* and *Phoenix dactylifera L*. endocarp extract was dissolved in distilled water at the concentration of 1 mg/mL and a filter paper having pore size 0.2 μm used to filter the solution. A total of 10‐μL filtered sample was subjected to LC–MS analysis. A Hewlett‐Packard series 1100 LC–MS system equipped with reverse phase C18 column with pore size 5 μm, with solvent gradient rate set at 5% to 100% range CH_3_CN for 23 min, flow rate set at 0.7 mL/min and UV detection. The mass data with low resolution recorded in positive mode. Nitrogen gas temperature was set at 350°C while drying gas flow rate was set at 10.01/min. Different voltage setting was applied as capillary voltage at 4000 V and ESI voltage at 6.0 kV. Capillary temperature was set at 200°C, and auxiliary and sheath gas pressure was set at 5 units and 70 lb/in.^2^ (Nabeshima et al. 2022).

### 7.2 Fourier Transform Infra‐Red Spectrum Analysis

FT‐IR used to quantify various functional groups in *Phoenix dactylifera* and *Phoenix dactylifera L*. endocarp extract. For the identification of functional groups, the sample has a dipole moment to indicate infrared activity. The sample was distributed on a disc of potassium bromide. The FT‐IR IRSpirit + QATR‐S System was used to perform FT‐IR analysis with spectrophotometer range of 400–4000 cm^−1^ and resolution of 2 cm^−1^. The results were labeled as transmittance versus wave number (Vogt et al. 2019).

### 7.3 UV–Visible Analysis

UV–Visible analysis of *Phoenix dactylifera* and *Phoenix dactylifera* *L*. endocarp extract was carried out in order to measure the reflectance of the transition state of highly conjugated biomolecules in the sample. The absorbance at 300–700 nm was determined by using a UV–Visible spectrophotometer in order to measure the absorbed light of biomolecules from the electromagnetic portion of the spectrum (Habchi et al. 2022).

## 8 In Silico Analysis

### 8.1 Pharmacokinetic Evaluation

When developing a new therapeutic medication, knowledge of pharmacokinetics' fate of a molecule in the body is essential. It is usually accomplished using individual indices called Absorption, Distribution, Metabolism, Excretion, and Toxicity (ADMET) components. Swiss ADME (http://www.swissadme.ch/), an online tool, was used to determine the pharmacokinetics of the compounds identified from LC/MS of both extracts of *Phoenix dactylifera* and *Phoenix dactylifera L*. (Pradeep et al. 2021).

### 8.2 Molecular Docking Analysis

The chemical structures of the compounds were retrieved from PubChem in SMILES format and subsequently converted into 3D mol files using ACD/ChemSketch for further processing. Molecular docking analysis was carried out to investigate the anti‐aging potential of phytochemicals from *Phoenix dactylifera* and *Phoenix dactylifera L*. possessing log Kp values below 6, suggesting favorable skin permeation abilities. The target proteins selected for dermatocosmetic evaluation included elastase (1RMZ), tyrosinase (5M8P), and hyaluronidase (1FCV), which were obtained from the RCSB Protein Data Bank www.rcsb.org/pdb in PDB format. Protein preparation involved the removal of water molecules and co‐crystallized ligands prior to docking studies. Standard preparation procedures were applied to both proteins and ligands before submitting the files to PyRx 1.1 with requirements of a DSL internet connection, a Windows 10 (latest Service Pack) 64‐bit operating system, 8 GB of RAM, 4 GB of GPU memory, and a screen resolution of 1024 × 768 pixels. Docking calculations were performed using a manually defined grid box of 50 Å along the X, Y, and Z coordinates. The binding energies and intermolecular interactions of each ligand were analyzed, and the resulting docked complexes were further visualized and examined using Discovery Studio (Chikowe et al. 2024).

### 8.3 Statistical Analysis

The experimental data was analyzed using one‐way analysis of variance (*T*‐test) with the assistance of SPSS software for Windows (IBM SPSS). The objective was to analyze and compare the outcomes of both the control group and the treatment group, as well as within the different subgroups of the treatment group. The data presented as the mean ± SEM. *p*‐value ≤ 0.05 was considered statistically significant (Quinty et al. 2022).

## 9 Results and Discussion

### 9.1 Total Phenolic and Flavonoid Content

The total phenolic content (TPC) and total flavonoid content (TFC) of *Phoenix dactylifera* increased progressively with rising concentrations (Table 1). At 25 mg/mL, TPC and TFC were 33.13 ± 1.3 mg GAE g^−1^ and 9.61 ± 1.2 mg CE g^−1^, respectively, which increased to 54.2 ± 1.81 mg GAE g^−1^ and 15.5 ± 1.7 mg CE g^−1^ at 50 mg/mL. At 75 mg/mL, further elevation was observed with TPC reaching 68 ± 1.56 mg GAE g^−1^ and TFC 28.481 ± 3.04 mg CE g^−1^, while the highest values were recorded at 100 mg/mL with TPC of 92 ± 1.15 mg GAE g^−1^ and TFC of 39.2 ± 0.11 mg CE g^−1^. Similarly, *Phoenix dactylifera* *L*. showed an increasing trend in both TPC and TFC with increasing concentration. At 25 mg/mL, TPC and TFC were 31.3 ± 2.1 mg GAE g^−1^ and 6.1 ± 1.3 mg CE g^−1^, respectively, rising to 57.1 ± 1.23 mg GAE g^−1^ and 14.2 ± 1.21 mg CE g^−1^ at 50 mg/mL. At 75 mg/mL, TPC further increased to 64 ± 1.17 mg GAE g^−1^, accompanied by a TFC of 23.42 ± 2.1 mg CE g^−1^, indicating a concentration‐dependent increase in phenolic and flavonoid contents across both samples. Phenolic compounds are recognized for their skin anti‐aging benefits. Polyphenols also possess strong anti‐inflammatory, antibacterial and UV‐protective activities. Flavonoids, which include flavones, flavonols, isoflavones, flavanones and chalcones are natural plant‐derived phenolic compounds with powerful antioxidant properties (Intharuksa et al. 2024). In a previous study, the methanol extract of marigold contained 74.8 mg TAE/g of total polyphenols and 85.6 mg RE/g of total flavonoids. The flavonoid level observed in the methanol extract of the whole Korean marigold plant was higher than the 68.9 mg RE/g previously reported methanol extracts obtained from Thai marigold flowers (Rivas‐García et al. 2023). Similarly, another study found that ethanol extracts from 11 Chinese marigold cultivars contained flavonoid levels ranging between 28.6 and 93.3 mg RE/g. These variations suggest that differences in cultivar type, plant part used and extraction solvent may influence the flavonoid content reported across studies (Sultana et al. 2025). The total phenolic content (TPC) and total flavonoid content (TFC) of *T. stans* flower extracts varied depending on the solvent used, as shown (Abo Khaled et al. 2025). Ethanol produced the highest TPC, measuring 24.10 ± 2.07 mg GAE/g extract, which was significantly greater than the values obtained with deionized water and ethyl acetate. The hexane extract showed the lowest TPC at 6.90 ± 0.47 mg GAE/g extract. These findings suggest that extracts prepared with more polar solvents tend to contain higher levels of phenolic compounds. For flavonoid content, the ethyl acetate extract demonstrated the highest TFC at 205.11 ± 7.83 mg CE/g extract, followed by ethanol, hexane and water extracts (Maneerat et al. 2025). Ethyl acetate extracts contained the greatest amount of phenolic compounds, whereas dichloromethane extracts were particularly rich in flavonoids (Xiang et al. 2024). In another study reported that distilled water extracts of *T. stans* leaves contained 12.0 ± 2.1 g GAE/kg extract of phenolics and 1.2 ± 0.1 g QE/kg plant extract of flavonoids. Observed no major differences in the TPC of *C. ternatea* flower extracts prepared using ethanol, acetone or distilled water (El Mannoubi 2023). Variations in TPC and TFC among studies are likely related to differences in plant species and the chemical characteristics of the compounds extracted, which are strongly influenced by solvent polarity (Savic et al. 2026).

**TABLE 1:** Determination of phenolic, flavonoid and radical scavenging capacity.

	Concentrations	TPC mg GAE g‐1	TFC mg CE g‐1	DPPH %
*Phoenix dactylifera*	25 mg/mL	33.13 ± 1.3	9.61 ± 1.2	48.47% ± 1.9%
	50 mg/mL	54.2 ± 1.81	15.5 ± 1.7	61.19% ± 1.32%
	75 mg/mL	68 ± 1.56	28.481 ± 3.04	76.5% ± 2.3%
	100 mg/mL	92 ± 1.15	39.2 ± 0.11	81.2% ± 1.46%
*Phoenix dactylifera L*.	25 mg/mL	31.3 ± 2.1	6.1 ± 1.3	43.21% ± 1.29%
	50 mg/mL	57.1 ± 1.23	14.2 ± 1.21	64.75% ± 1.6%
	75 mg/mL	64 ± 1.17	23.42 ± 2.1	72.21% ± 2.42%
	100 mg/mL	82.3 ± 1.01	31.2 ± 2.01	89.16% ± 1.26%
Control^a^ 100 μg/mL	—	—	—	98.12% ± 1.12%

*Note:* The measurements represent the mean ± SE of three independent calculations.

^a^ Quercetin.

### 9.2 DPPH Radical Scavenging Activity

The antioxidant activity of *Phoenix dactylifera* was evaluated using the DPPH radical scavenging assay at different concentrations. The results showed a concentration‐dependent increase in antioxidant activity (Table 1). At 25 mg/mL, the extract exhibited 48.47% ± 1.9% DPPH inhibition, which increased to 61.19% ± 1.32% at 50 mg/mL. Further increases in concentration enhanced the scavenging activity to 76.5% ± 2.3% at 75 mg/mL and 81.2% ± 1.46% at 100 mg/mL. Similarly, *Phoenix dactylifera L*. demonstrated strong antioxidant potential with increasing concentrations. The DPPH scavenging activity was recorded as 43.21% ± 1.29% at 25 mg/mL, 64.75% ± 1.6% at 50 mg/mL, 72.21% ± 2.42% at 75 mg/mL, and 89.16% ± 1.26% at 100 mg/mL. The standard control at 100 μg/mL showed the highest antioxidant activity, with 98.12% ± 1.12% inhibition. These findings indicate that the antioxidant capacity of the extracts improved with increasing concentration and approached the activity of the control at higher doses. The *p*‐value for *Phoenix dactylifera L*. was 0.006, while for *Phoenix dactylifera* it was 0.015. Both values are lower than 0.05, indicating statistically significant results. Natural antioxidants play an important role in neutralizing free radicals thereby helping to reduce inflammation and slowing the aging process of the skin (Oyovwi et al. 2025). Several methods are commonly used to evaluate the antioxidant properties of natural products, including free radical scavenging, inhibition of lipid peroxidation and metal chelation assays. In the DPPH assay, antioxidants donate hydrogen atoms to DPPH radicals, converting them into stable hydrazine molecules and reducing free radical activity (Gulcin and Alwasel 2023). The antioxidant activities observed in the *Phoenix dactylifera* extracts in a previous study may be associated with their flavonoid and phenolic constituents. Phenolic compounds such as corilagin, ellagic acid, geraniin and quercetin present in the extract have previously been reported to exhibit strong antioxidant effects through DPPH radical scavenging (Tiranakwit et al. 2023). Similar findings have also been reported for *Phoenix dactylifera* peel extracts from other species, which showed high total phenolic and flavonoid contents together with significant antioxidant activities in assays such as ABTS, DPPH, superoxide scavenging, ferric reducing antioxidant power (FRAP), metal chelation and lipid peroxidation inhibition (Adli et al. 2022).

### 9.3 Antioxidant Enzymes Activities

The antioxidant enzyme activities of *Phoenix dactylifera* and *Phoenix dactylifera* *L*. were evaluated at different concentrations (25, 50, 75, and 100 mg/mL) by measuring peroxidase, catalase, and superoxide dismutase activities (Table 2). In *Phoenix dactylifera* , peroxidase activity increased progressively from 16.94 ± 1.94 IU/mg at 25 mg/mL to 35.15 ± 1.35 IU/mg at 100 mg/mL. Similarly, catalase activity rose from 4.69 ± 0.10 IU/mg to 15.17 ± 1.42 IU/mg, while superoxide dismutase activity increased from 8.13 ± 0.13 IU/mg to 26.45 ± 0.14 IU/mg with increasing concentration. In *Phoenix dactylifera* *L*., peroxidase activity was recorded as 11.52 ± 1.14 IU/mg at 25 mg/mL, increasing to 23.12 ± 1.30 IU/mg at 75 mg/mL, followed by a slight decrease to 19.5 ± 1.05 IU/mg at 100 mg/mL. Catalase activity showed a gradual increase from 2.21 ± 0.10 IU/mg at 25 mg/mL to 13.75 ± 0.32 IU/mg at 100 mg/mL. Likewise, superoxide dismutase activity increased steadily from 5.21 ± 1.98 IU/mg at 25 mg/mL to 24.45 ± 1.40 IU/mg at 100 mg/mL. Overall, both samples demonstrated concentration‐dependent enhancement in antioxidant enzyme activities, with *Phoenix dactylifera* generally exhibiting higher enzyme activities compared to *Phoenix dactylifera* *L*. *Phoenix dactylifera* showed a statistically significant effect on antioxidant enzyme activities with a *p*‐value of 0.0184 (*p* < 0.05). In contrast, *Phoenix dactylifera* *L*. exhibited a non‐significant variation in enzyme activities with a *p*‐value of 0.0762 (*p* > 0.05). Skin aging is strongly linked to oxidative stress in dermal fibroblasts. Therefore, compounds capable of protecting fibroblasts from oxidative injury may serve as promising anti‐aging agents in cosmeceutical formulations (Biswas et al. 2022). Results from a previous study of MTT assays and bioenergetic analysis demonstrated that Centella extract markedly reduced H_2_O_2_‐induced cytotoxicity in fibroblasts, with protective effects. The observed protection appeared to be associated with an enhanced ability of fibroblasts to neutralize reactive oxygen species (ROS) (Fadzli et al. 2025). Catalase (CAT) primarily detoxifies high concentrations of H_2_O_2_, whereas glutathione peroxidase 1 (GPx1) removes lower levels of H_2_O_2_ (Liu et al. 2022). Superoxide dismutase enzymes, including cytosolic SOD1 and mitochondrial SOD2, are responsible for converting superoxide radicals into H_2_O_2_, which is subsequently eliminated by catalase and other peroxidases (Sang et al. 2022). Under oxidative stress conditions induced by H_2_O_2_, pretreatment with *Centella* extract elevated CAT, GPx1, and SOD1 expression. The increased expression of these antioxidant enzymes likely improved the capacity of fibroblasts to remove ROS, thereby reducing oxidative damage and preventing cell death (Ali et al. 2026). In rat models of liver injury, *Centella* extract reduced hepatotoxicity by elevating catalase, SOD, and GPx levels in hepatic tissues (Ferah Okkay et al. 2022). Ethanolic extracts of *Centella asiatica* enhanced liver function in hyperlipidemic hamsters through increased SOD and GPx expression. In diabetic rats, aqueous extracts improved hippocampal function by inducing catalase, SOD, and GPx expression in brain tissues (Yusuf et al. 2022). In models of ischemic stroke, ethanolic extracts helped protect the brain from damage by bringing back glutathione levels and boosting the activities of catalase, SOD, and GPx (Sivagurunathan et al. 2025). Nrf2 acts as a sensor for oxidative stress and, upon activation, translocates to the nucleus, where it binds to ARE sequences, promoting the transcription of cytoprotective genes such as CAT, GPx, and SOD (Kumar and Ramanarayanan 2025). Previous studies have shown that *Centella* extracts can activate the Nrf2/ARE pathway in several models of oxidative‐stress‐related disorders. Water extracts of *Centella asiatica* have been reported to reduce oxidative stress and stimulate Nrf2/ARE signaling in primary neurons and animal models of cognitive impairment, leading to improved neuronal function and cognition (Hambali et al. 2024).

**TABLE 2:** Enzymatic activities of *Phoenix dactylifera* and *Phoenix dactylifera* L.

	Concentration	Peroxidase (IU/mg)	Catalase (IU/mg)	Superoxide dismutase (IU/mg)
*Phoenix dactylifera*	25 mg/mL	16.94 ± 1.94	4.69 ± 0.1	8.13 ± 0.13
	50 mg/mL	24.43 ± 0.17	5.78 ± 1.34	14.05 ± 1.24
	75 mg/mL	29.31 ± 0.13	9.34 ± 2.45	21.25 ± 1.14
	100 mg/mL	35.15 ± 1.35	15.17 ± 1.42	26.45 ± 0.14
*Phoenix dactylifera L*.	25 mg/mL	11.52 ± 1.14	2.21 ± 0.1	5.21 ± 1.98
	50 mg/mL	19.56 ± 1.23	4.54 ± 0.14	10.13 ± 0.23
	75 mg/mL	23.12 ± 1.3	6.21 ± 1.4	19.42 ± 0.23
	100 mg/mL	19.5 ± 1.05	13.75 ± 0.32	24.45 ± 1.4

*Note:* The measurements represent the mean ± SE of three independent calculations.

### 9.4 Antiaging Analysis

The anti‐aging potential of *Phoenix dactylifera* and *Phoenix dactylifera L*. was evaluated through elastase, hyaluronidase, and tyrosinase inhibition assays at different concentrations (25–100 mg/mL) (Table 3). In *Phoenix dactylifera* , elastase inhibition increased from 21.54% ± 0.51% at 25 mg/mL to 49.69% ± 1.15% at 100 mg/mL. Similarly, hyaluronidase inhibition rose from 31.3% ± 1.52% to 51.65% ± 1.49%, while tyrosinase inhibition increased from 30.15% ± 1.32% to 50.41% ± 1.32% with increasing concentration. In *Phoenix dactylifera L*., elastase inhibition was recorded as 18.13% ± 0.34% at 25 mg/mL and increased to 45.5% ± 0.10% at 100 mg/mL. Hyaluronidase inhibition also showed a concentration‐dependent increase, ranging from 28.1% ± 1.81% at 25 mg/mL to 48.2% ± 0.19% at 100 mg/mL. Overall, both extracts demonstrated dose‐dependent inhibitory activities against skin‐aging enzymes, with *Phoenix dactylifera* exhibiting comparatively higher inhibitory effects than *Phoenix dactylifera L*. The control at 10 μg/mL showed the highest inhibitory activities, with elastase inhibition of 94.67% ± 1.32%, hyaluronidase inhibition of 99.12% ± 0.98% and tyrosinase inhibition of 94.12% ± 1.27%, indicating strong enzyme inhibitory potential compared to the tested extracts. Statistical analysis demonstrated that both *Phoenix dactylifera and Phoenix dactylifera L*. exhibited significant inhibitory activities against the tested skin‐aging enzymes. The *p*‐values obtained for *Phoenix dactylifera* (0.0286) and *Phoenix dactylifera L*. (0.0141) were both below 0.05, indicating statistically significant differences among the tested concentrations. The findings revealed that all extracts *of T. stans* contained important phytochemical constituents, including flavonoids, phenolics, terpenoids, steroids, and alkaloids. These secondary metabolites possess diverse chemical structures that contribute significantly to their antioxidant and anti‐aging effects (Wichayapreechar et al. 2024). Previous studies have reported that flavonoids and phenolic acids present in ethanolic, methanolic, deionized water, and ethyl acetate extracts exhibit strong antioxidant potential. Natural antioxidant compounds can neutralize free radicals and thereby suppress the activity of tyrosinase, elastase, and hyaluronidase enzymes, which are closely associated with wrinkle formation and skin aging (Kumar et al. 2025). Among the tested extracts, the absolute ethanolic extract of *T. stans* demonstrated the greatest anti‐aging potential based on anti‐collagenase, anti‐elastase, and anti‐hyaluronidase activities. This enhanced activity may be related to the presence of phytochemicals such as flavonoids, phenolics, terpenoids, and alkaloids, together with its strong antioxidant properties resulting from high total phenolic and flavonoid contents (Tammasorn et al. 2023). In addition, the deionized water and ethyl acetate extracts also showed notable anti‐aging activities, despite exhibiting antioxidant activities higher than that of ethanolic extract. These observations suggest that the anti‐aging effects of *T. stans* are not solely dependent on antioxidant activity but may also involve other bioactive compounds or synergistic interactions among the phytochemical constituents present in the extracts (Gupta and Jain 2024).

**TABLE 3:** Antiaging analysis of *Phoenix dactylifera* and *Phoenix dactylifera* L.

	Concentration	Elastase inhibition (%)	Hyaluronidase inhibition (%)	Collagenase inhibition (%)
*Phoenix dactylifera*	25 mg/mL	21.54 ± 0.51	31.3 ± 1.52	32.15 ± 1.09
	50 mg/mL	38.33 ± 0.96	33.23 ± 1.2	39.76 ± 1.67
	75 mg/mL	41.6 ± 0.75	45.52 ± 1.94	47.14 ± 2.13
	100 mg/mL	49.69 ± 1.15	51.65 ± 1.49	58.41 ± 1.98
*Phoenix dactylifera L*.	25 mg/mL	18.13 ± 0.34	28.1 ± 1.81	27.01 ± 0.66
	50 mg/mL	27.1 ± 1.71	31.14 ± 2.1	36.08 ± 1.76
	75 mg/mL	39.5 ± 1.32	39.32 ± 1.3	42.5 ± 1.31
	100 mg/mL	45.5 ± 0.1	48.2 ± 0.19	49.1 ± 0.65
Control	10 μg/mL	94.67 ± 1.32^a^	99.12 ± 0.98^b^	89.12 1.87^c^

*Note:* The measurements represent the mean ± SE of three independent calculations.

^a^ Elafin.

^b^ Sodium aurothiomalate.

^c^ EDTA.

## 10 Analytical Characterization

### 10.1 Liquid Chromatography Mass Spectrophotometry

**FIGURE 1:**
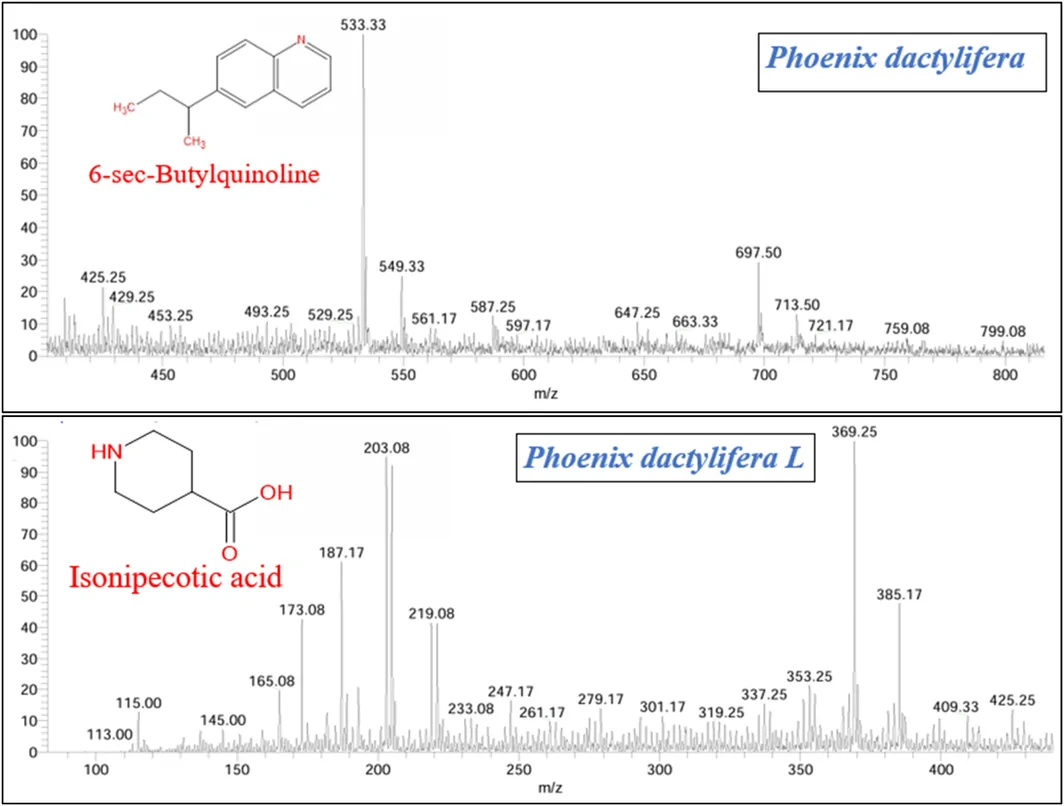
Chromatogram of mass spectrometry of *Phoenix dactylifera* and *Phoenix dactylifera L*.

The given data provides information on various compounds identified through mass spectrometry, including their *m*/*z* (mass‐to‐charge ratio), intensity, retention time, and molecular mass. Several biologically active compounds were found in both *Phoenix dactylifera* and *Phoenix dactylifera L*. by comparing the fragmentation patterns (i.e., product ions) obtained by LC–MS analysis with the molecular (precursor) ion (Figure 1).

The LCMS was subsequently compared with the published literature, databases (PDB), and computer archives (NIST and European Mass Data Bank) for reference substances outcomes (Table 4). LC–MS/MS profiling revealed the presence of a diverse range of bioactive metabolites belonging to different chemical classes, including alkaloids, flavonoids, glycosides, saponins, quinolines, vitamins, pyridine derivatives and phenolic compounds. Among these, particular attention was given to the phenolic acids due to their well‐established antioxidant and therapeutic potential. Several important phenolic acid derivatives were identified, including caffeic acid, benzoic acid, glutaric acid derivatives, pimelic acid derivatives, sulfonic acid derivatives and ritalinic acid‐related compounds. The detection of caffeic acid is especially significant because it is a known phenolic antioxidant associated with free radical scavenging, anti‐inflammatory and antimicrobial activities. Likewise, benzoic acid and related aromatic acid derivatives contribute to antioxidant stability and preservation properties. Among the detected metabolites, telmisartan glucuronide was identified at *m*/*z* 689.30 with a molecular mass of 690.30 and retention time of 3.25 min, indicating the presence of glucuronidated metabolites. 4‐Dodecylmorpholine (*m*/*z* 254.10) and oleic diethanolamide (*m*/*z* 368.30) represented lipid‐associated and alkanolamide compounds that may contribute to membrane‐associated biological activities. Oleic diethanolamide an alkanolamide derivative, is known for its surfactant and anti‐inflammatory potential. Several nitrogen‐containing bioactive compounds were also detected, including bicuculline an alkaloid with *m*/*z* 366.30 and Oxoglaucine an aporphine alkaloid detected at *m*/*z* 350.30. The LC–MS/MS profile also identified aromatic and benzenoid compounds such as benzamide and N‐(3‐chlorophenyl)‐3‐methoxybenzamide. These compounds are structurally related to aromatic amides and may contribute to antimicrobial and antioxidant activities. Dihydrocapsaicin, a capsaicinoid compound detected at *m*/*z* 348.30, further indicates the occurrence of bioactive secondary metabolites with potential anti‐inflammatory and analgesic properties. Kynurenic acid and xanthurenic acid, both biologically important metabolites associated with tryptophan metabolism, were also identified, indicating the presence of neuroprotective and antioxidant‐related metabolites. In addition to phenolic acids, flavonoid compounds were also detected, particularly flavone, which exhibited *m*/*z* 814.30 and a retention time of 4.53 min. Flavonoids are among the most important classes of polyphenolic compounds and are widely recognized for their antioxidant, antimicrobial, anti‐inflammatory and anticancer activities. The metabolomic analysis further demonstrated the presence of biologically important glycosides and saponins. Eleutheroside E, a glycoside compound, was identified at *m*/*z* 742.30, while madecassoside and furostane derivatives were detected at *m*/*z* 974.50 and 870.50, respectively. Several additional compounds of pharmacological importance were identified, including indole‐3‐pyruvate, myrtenol, sarcosine derivatives, leucine encephalin, nicotinate mononucleotide, senkirkine, renardine, Benzoquinoline and pantothenic acid. Indole‐3‐pyruvate is associated with indole metabolism and antioxidant activities, whereas myrtenol is a monoterpenoid known for antimicrobial and anti‐inflammatory properties. The simultaneous occurrence of flavonoids and phenolic acid derivatives strongly correlates with the higher total phenolic content observed during phytochemical analysis. The abundance of these polyphenolic metabolites indicates that the extract possesses considerable reducing power and radical scavenging capacity, which may contribute significantly to its biological and therapeutic potential. LC–MS/MS analysis in positive‐ion mode [M − H]^+^ revealed the presence of nine compounds including 5‐(7‐Methyloctethanol), Dodecane, Pyrocoll, Methyl hydroxysterpurate ethylidene acetal, 3β‐hydroxy‐4β‐methylfusida‐17Terezine E and Paeciloquin. Similarly, the negative‐ion mode [M − H]^−^ chromatograms also showed nine metabolites, including complex hydroxy‐spiro compounds, Fensulfothion, 8‐Hydroxyloxapine, Gilvsin A, Sitosterol, Lansoprazole, Isoxsuprine, 2‐Aminooctadecane, and Dehydroevodiamine.

**TABLE 4:** LCMS analysis of *Phoenix dactylifera L. and Phoenix dactylifera*.

Compound	Class	*m*/*z*	Intensity	Retention time	Molecular mass
Telmisartan glucuronide^a^	—	689.30−	214.83, 378.00, 586.42	3.25	690.30
4‐Dodecylmorpholine^b^	—	254.10−	110.92, 86.92, 124.08	1.27	255.10
Oleic diethanolamide^b^	Alhanolamide	368.30+	185.00, 310.25, 351.25	3.03	369.30
Bicuculline^b^	Alkaloids	366.30+	205.08, 279.08, 308.25	2.90	367.30
Benzamide^b^		384.30+	221.08, 245.08, 313.17	3.48	385.30
Oxoglaucine^b^	Aporphine	350.30+	110.92, 137.00, 195.00	2.71	351.30
N‐(3‐chlorophenyl)‐3‐methoxybenzamide^b^	Benzenoid	260.30+	161.00, 201.08, 135.00	2.02	261.30
Dihydrocapsaicin^a^	Capsaicinoid	348.30+	225.08, 251.08, 281.17	2.61	349.30
Kynurenic acid^b^	Carboxylic Aid	332.30+	235.08, 261.08, 111.00	2.50	333.30
Apicidin^a^	Cyclic tetrapeptides	622.30−	204.00, 300.00, 315.08	2.84	623.30
Flavone^a^	Flavonoids	814.30−	537.33, 559.33, 577.42	4.53	815.30
Phosphatidylcholine^a^	Glycerophospholipids	830.50−	255.25, 391.25, 553.33	4.68	831.50
Eleuthero side E^a^	Glycoside	742.30−	277.25, 391.17, 447.33	4.09	743.30
Indole‐3‐pyruvate^b^	Indole	202+	112.92, 142.92, 133.00	1.64	203.00
Myrtenol^a^	Monoterpenoid	316.30+	155.00, 197.00, 247.08	2.28	317.30
Sarcosine^b^	N‐alkylglycine	382.30+	178.25, 247.08, 345.25	3.35	383.30
leucine encephalin^a^	Opioid peptides	554.30−	164.92, 206.92, 411.25	2.60	555.30
2‐(Methylthio)benzothiazole^a^	Organic sulfide	180−	71.00, 91.08, 163.00	0.54	181.00
l‐Phenylalanine, n‐heptafluorobutyryl‐, pentadecyl ester^a^	Perfluoroacylated amino acid esters.	570.30−	187.08, 223.00, 241.00	2.61	571.30
Glutaric acid^a^	Phenolic ester	292.20−	96.92, 125.00, 149.00	1.70	293.20
Pimelic acid^a^		326.30−	97.00, 125.00, 137.00	1.80	327.30
Isonipecotic acid^a^		280.20−	83.00, 96.75, 111.08	1.40	281.20
Sulfonic acid^a^		328.20−	99.08, 125.00, 139.08	1.91	329.20
Ritalinic acid^a^		362.20−	112.92, 125.00, 227.00	2.06	363.20
Isonipecotic acid, N‐(3‐methylbutyryl)‐, dodecyl ester^a^		380.30+	200.92, 345.25, 349.17	3.27	381.30
Benzoic acid^b^		178−	71.08, 74.92, 116.92	0.48	179.00
Carbonic acid (monoamide, N‐pentyl‐, hexyl ester)^b^		214.1−	179.00, 161.00, 64.58	0.85	215.01
Caffeic acid ^a^		179+	120.00, 144.92, 162.92	0.24	180.00
Sarcosine N‐(4‐methylbenzoyl)‐phenyl ester^b^		276.30+	91.00, 175.17, 221.08	2.12	277.30
2‐Cyclohexylamino‐5‐nitropyridine^b^	Pyridine	220.30+	81.00, 135.00, 175.08	1.91	221.30
Nicotinate mononucleotide^b^	Pyridine nucleotide	334.30+	179.08, 195.00, 251.25	2.51	335.30
Senkirkine^a^	Pyrrolizidine alkaloid	364.30−	104.83, 125.00, 167.00	2.11	365.30
Renardine^a^		364.30+	184.92, 203.00, 220.33	2.80	365.30
6‐sec‐Butylquinoline^a^	Quinolines	184+	108.75, 142.92, 157.00	0.54	185.00
Xanthurenic acid^b^		204+	116.83, 122.08, 130.92	1.72	205.00
Benzoquinoline^b^	Quinone	218−	73.00, 87.92, 100.92	1.10	219.00
Madecassoside^b^	Saponin	974.50+	519.50, 681.50, 697.42	4.66	975.50
Furostane^b^		870.50+	533.33, 593.33, 615.42	4.07	871.50
Mediagenic acid^b^		662.30+	383.25, 501.25, 619.42	3.80	663.30
Pantothenic acid^b^	Vitamin	218.20+	109.00, 149.00, 191.08	1.80	219.20

^a^ *Phoenix dactylifera* L.

^b^ *Phoenix dactylifera*.

These compounds were detected at different retention times and *m*/*z* values, indicating the chemical diversity of the analyzed extract (Almalki et al. 2023). Polyphenols are an important group of plant secondary metabolites characterized by polyphenolic structures and well known for their biological activities, particularly antioxidant effects (Alsuhaymi et al. 2023). Phenolic acid compounds were identified in both the seed and flesh, including 4‐hydroxybenzoic acid, which has also been previously detected in mesocarp extracts of the sugar date palm *Phoenix sylvestris* . Additionally, gentisic acid was found exclusively in the seeds. This compound also occurs naturally in the roots of the genus Gentiana as well as in the fungus *Penicillium brevicompactum* (Abdelghffar et al. 2022). Gentisic acid has also demonstrated antioxidant properties, helping to counteract oxidative stress induced chemically, enzymatically and by ultraviolet (UV) radiation. Another compound identified was vanillic acid, a flavor‐related molecule and an oxidized derivative of vanillin (Baig et al. 2026). Vanillic acid has been reported to exert anti‐inflammatory effects by inhibiting the production of pro‐inflammatory cytokines and suppressing the activation of nuclear factor‐kappa B NF‐κB and caspase‐1 (Wani et al. 2025). Caffeic acid (3,4‐dihydroxycinnamic acid), the most important class of the hydroxycinnamic acid group is used as an antioxidant, anti‐inflammatory and antibacterial agent (Alam et al. 2024). Ferulic acid and sinapic acid were only found in flesh samples. Sinapic acid (3,5‐dimethoxy‐4‐hydroxycinnamic acid) is a common dietary component widespread in cereals, fruits and vegetables. Its derivatives have substantial bioactivities; for instance, 4‐vinylsyringol (a sinapic acid derivative product) has significant antioxidant and anticancer properties and inhibits carcinogenesis and the generation of inflammatory cytokines (El‐Gendy et al. 2026).

### 10.2 FTIR Analysis

**FIGURE 2:**
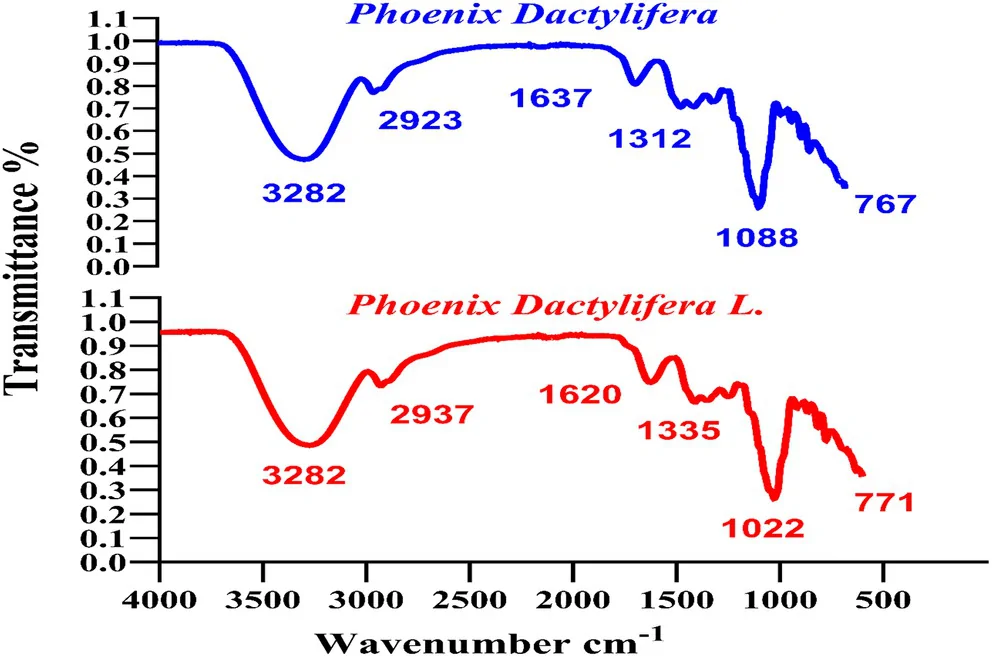
FTIR spectrum of *Phoenix dactylifera L*. and *Phoenix dactylifera*.

The FTIR analysis of samples of *Phoenix dactylifera* *L*. and *Phoenix dactylifera* showed some similarities and differences in their peak values, which correspond to various functional groups in organic molecules (Figure 2). Both *Phoenix dactylifera* *L*. and *Phoenix dactylifera* exhibit a strong peak at 3282 cm^−1^ combined with the stretching vibration of the OH group commonly found in alcohols and phenols. For the O–H stretching vibration, *Phoenix dactylifera* *L*. displays a peak at 2937 cm^−1^, which is indicative of carboxylic acids, while *Phoenix dactylifera* represents a similar peak at 2923 cm^−1^. The NH stretching vibration in *Phoenix dactylifera* L. appears at 1620 cm^−1^, showing the presence of amino acids or amine‐containing compounds, while in *Phoenix dactylifera* , this peak is slightly shifted to 1637 cm^−1^. The CH_3_ rocking motion is represented by a peak at 1335 cm^−1^ in *Phoenix dactylifera* *L*., which could be assigned to organic phosphorus compounds, while in *Phoenix dactylifera* , this peak is at 1312 cm^−1^. The C=C stretching vibration indicative of hydrocarbons is analyzed at 1022 cm^−1^ in *Phoenix dactylifera* *L*. and at 1088 cm^−1^ in *Phoenix dactylifera* . Finally, the C–H deformation is shown at 771 cm^−1^ in *Phoenix dactylifera* *L*. and at 767 cm^−1^ in *Phoenix dactylifera* , which indicates secondary free alcohols. A broad absorption band at 3310 cm^−1^ is assigned to O–H stretching vibrations, which are linked to hydroxyl and carboxyl functional groups present in the juice of *Phoenix Dactylifera* . The peak at 2925 cm^−1^ corresponds to C–H stretching vibrations in hydrocarbons and aldehydes associated with the sugar backbone (Mahdi et al. 2023). The band observed at 1630 cm^−1^ is related to the asymmetric stretching of carboxylate groups (COO^−^), indicating the presence of pectin compounds in extract (Obode et al. 2025). The signal at 1591 cm^−1^ may be attributed to C–H and O–H bending vibrations originating from glucose and fructose molecules (Habchi et al. 2022). Meanwhile, the strong absorption peak at 1026 cm^−1^ is likely associated with C–O and C–C stretching vibrations in primary alcohols and glycosidic bonds within polysaccharides, confirming the presence of polymeric carbohydrate structures. These spectral features are important for the identification and characterization of pectins and other polysaccharides in plant‐based materials. Overall, no significant differences were observed in this region across different extraction temperatures, suggesting that the polysaccharide backbone remains structurally stable under the processing conditions used (Karunanithi and Varadappan 2025).

### 10.3 UV–Visible Analysis

**FIGURE 3:**
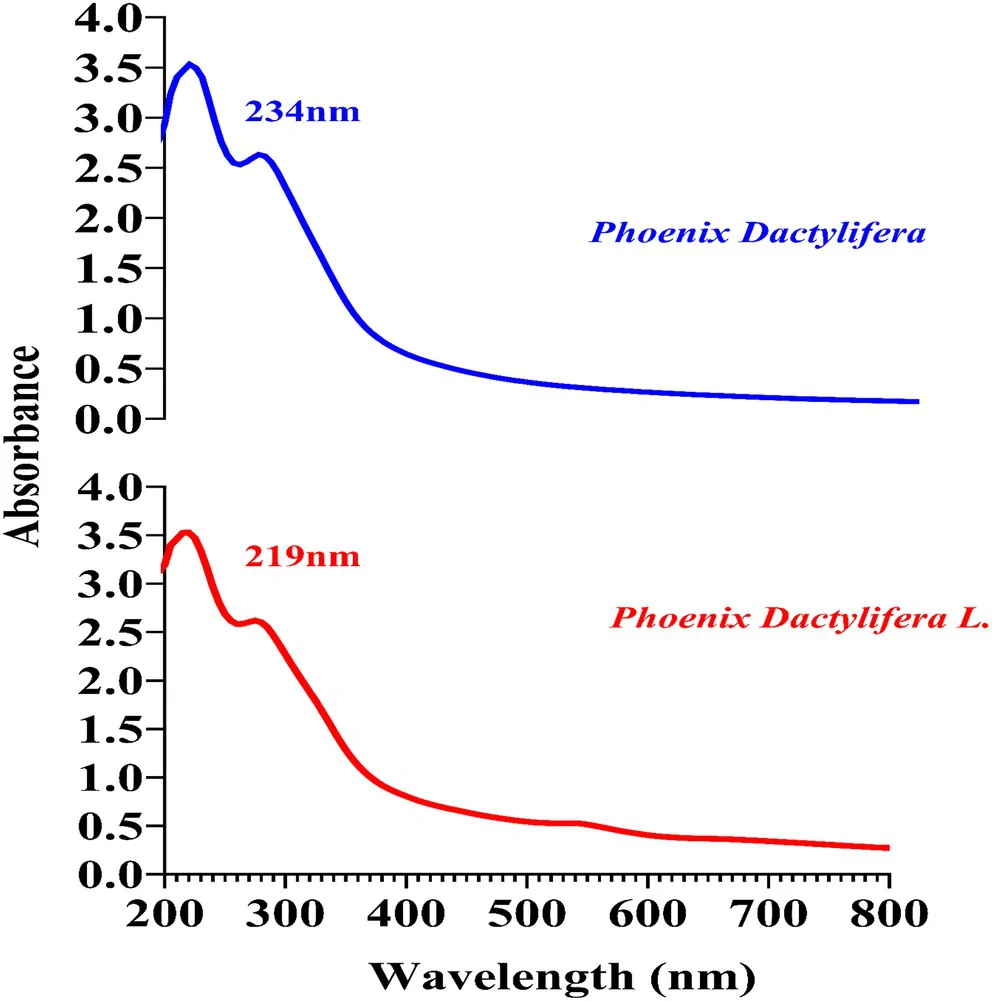
UV–Visible spectrum of *Phoenix dactylifera* and *Phoenix dactylifera L*.

The 200–800 nm range was used for the *Phoenix dactylifera* and *Phoenix dactylifera L*. UV–Vis response that was seen (Figure 3). There were only peaks in the UV–vis absorption spectra that were detected at wavelengths of 234 nm which are associated with phenolic‐like flavonoids, tannins, and phenolic acids‐based phytochemicals. The development of peaks is also visually evident in the FTIR spectra. In the case of *Phoenix dactylifera L*., absorption bands in the 219 nm were seen; however, in the case of intensity, the region considerably decreased, indicating an interaction between phytoconstituent and solvent. The UV–Vis spectral analysis of the date samples revealed several distinct absorption maxima at 220, 260, 470, 500, and 600 nm, with corresponding absorbance values of 0.42, 0.465, 0.966, 1.24, and 1.11. Absorption in the UV range, particularly near 220 and 260 nm, is commonly associated with phenolic and flavonoid compounds due to their aromatic ring systems (Ettadili et al. 2023). For example, phenolic acids such as gallic acid typically exhibit strong absorption around 220 nm, while flavonoids and catechin‐like compounds often show notable peaks near 260 nm. The peak observed at 470 nm may be linked to the presence of carotenoids. Compounds such as β‐carotene, lutein, and zeaxanthin generally absorb light within the 400–500 nm region, with a characteristic maximum around 470 nm (Raza et al. 2022). The absorption at 500 nm also falls within the typical range for carotenoids and may additionally indicate the presence of anthocyanins (Patil et al. 2023). Similarly, the absorption band at 600 nm is also characteristic of anthocyanins, particularly when they exist in neutral or slightly alkaline conditions, where their spectral properties can shift toward longer wavelengths (Maryam et al. 2025). The relatively high absorbance values at 470 and 500 nm indicate a substantial presence of carotenoid and anthocyanin pigments, both of which are recognized for their antioxidant activity. These findings are in agreement with previous studies reporting significant levels of phenolic and flavonoid‐related compounds in dates (Ahmad et al. 2024).

### 10.4 In Silico Pharmacokinetics Evaluation

**FIGURE 4:**
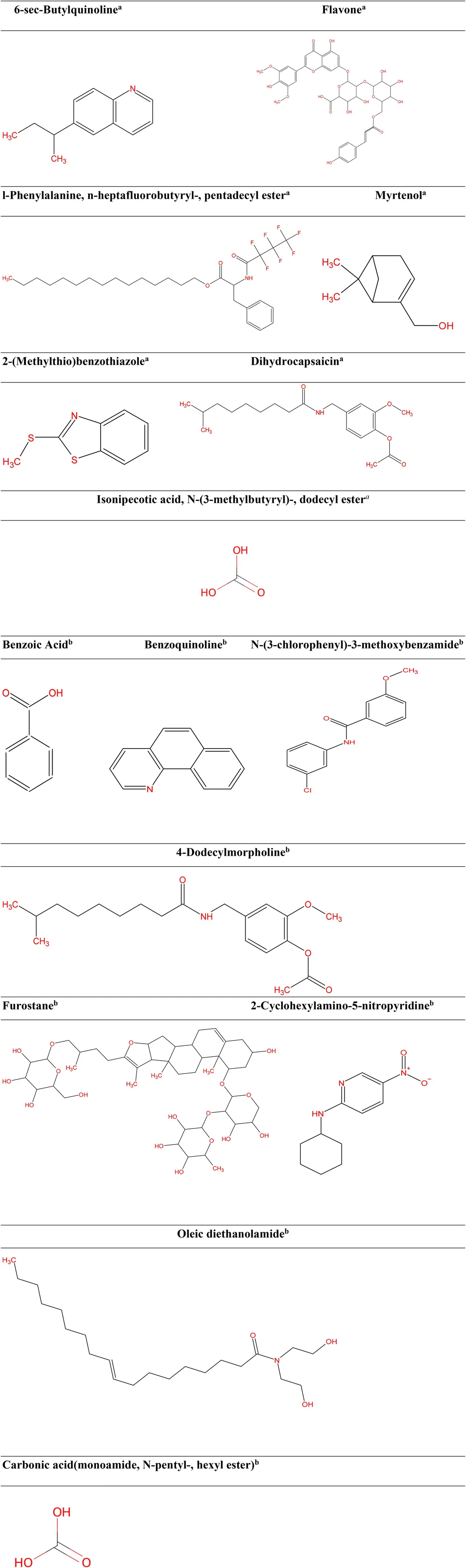
Structure of phytochemicals with high skin permeation.

In *Phoenix dactylifera* L., pharmacokinetic analysis of phytochemicals: 6‐sec‐Butylquinoline, phosphatidylcholine, flavone, l‐phenylalanine, *n*‐heptafluorobutyryl‐pentadecyl ester, myrtenol, 2‐(methylthio)benzothiazole, dihydrocapsaicin, isonipecotic acid, N‐(3‐methylbutyryl) and dodecyl ester—all these phytoconstituents have shown skin permeability and GI absorption and follow Lipinski's rules (Table 5, Figure 4). The main route of anti‐aging action is skin permeability; therefore, only skin‐permeable phytochemicals were selected and subsequently carried forward for molecular docking analysis. Among these phytochemicals, phosphatidylcholine (121.00 TPSA) has the highest skin permeability with a LogKp value of −0.27, shows inhibition of P‐Gp substrate, and has a bioavailability score of 0.55. While in *Phoenix dactylifera*, pharmacokinetic evaluation of phytochemicals, including benzoic acid, carbonic acid, benzoquinoline, 4‐dodecylmorpholine, furostane, oleic diethanolamide, N‐(3‐chlorophenyl)‐3‐methoxybenzamide, and 2‐cyclohexylamino‐5‐nitropyridine, has shown high skin permeability and high GI absorption, with a bioavailability score of 0.55 or, in some cases, ≥ 0.55. These phytochemicals have a TPSA range of 12.45 to 70.74, and all of these phytochemicals show inhibition of P‐Gp substrate, underscoring retention in the CNS. Most of the selected compounds intended for dermatocosmetic applications demonstrated favorable ADME profiles, including low gastrointestinal absorption, lack of blood–brain barrier (BBB) permeability and being substrates of P‐glycoprotein (Fatoki et al. 2023). Their log Kp values were also comparable to standard compounds such as kojic acid (−7.62 cm/s) and quercetin (−7.05 cm/s), indicating strong dermal retention and minimal systemic exposure, which in turn suggests a reduced risk of side effects. Skin permeability (Kp) refers to the rate at which a compound passes through the stratum corneum, the outermost layer of the epidermis (Chandrawati et al. 2025). A higher log K_ow (octanol–water partition coefficient) reflects greater lipophilicity and is commonly associated with enhanced skin penetration potential. Consequently, more lipophilic substances tend to accumulate within the lipid‐rich layers of the skin, making them particularly suitable for cosmetic use. In addition, low gastrointestinal absorption is generally advantageous for topical cosmetic ingredients, as it limits systemic uptake and thereby reduces potential health concerns (Abbassi et al. 2025).

**TABLE 5:** Pharmacokinetic evaluation of *Phoenix dactylifera L*. and *Phoenix dactylifera* derived phytochemicals.

Phytochemical	TPSA Å^2^	Solubility (class)	Log Kp cm/s	ESOL Log S	GI absorption	BBB parameant	P‐gp substrate	CyPIA2	Lipinskins	Consensus Log Po/w	Bioavailability scores
6‐sec‐Butylquinoline^a^	12.89	Soluble	−4.68	−3.82	High	Yes	No	Yes	Yes	3.40	0.55
Caffeic acid ^a^	77.76	Soluble	−6.58	−1.89	High	No	No	No	Yes	0.93	0.56
Phosphatidylcholine^a^	121.00	Insoluble	−0.27	−11.36	Low	No	Yes	No	Yes	9.10	0.55
Flavone^a^	30.21	Moderately soluble	−5.13	−4.09	High	Yes	No	Yes	Yes	3.18	0.55
Eleuthero side E^a^	254.14	Soluble	−11.81	−2.95	Low	No	Yes	No	No	−1.15	0.17
Apicidin^a^	138.84	Moderately soluble	−6.97	−5.84	High	No	Yes	No	No	3.16	0.17
l‐Phenylalanine, *n*‐heptafluorobutyryl‐, pentadecyl ester^a^	55.40	Poorly soluble	−1.89	−8.99	Low	No	No	No	No	8.84	0.17
Telmisartan glucuronide^a^	169.16	Poorly soluble	−6.71	−7.27	Low	No	Yes	Yes	No	3.97	0.11
Glutaric acid^a^	74.60	Soluble	−7.31	−0.21	High	No	No	No	Yes	0.01	0.85
Myrtenol^a^	20.23	Soluble	−4.94	−2.75	High	Yes	No	No	Yes	2.40	0.55
Pimelic acid^a^	74.60	Very soluble	−6.84	−0.82	High	No	No	No	Yes	0.75	0.85
Isonipecotic acid^a^	49.33	Highly soluble	−9.25	1.35	High	No	No	No	Yes	−0.28	0.55
2‐(Methylthio)benzothiazole^a^	66.43	Soluble	−5.17	−3.49	High	Yes	No	Yes	Yes	2.88	0.55
Dihydrocapsaicin^a^	58.56	Moderately soluble	−5.02	−4.02	High	Yes	No	Yes	Yes	3.70	0.55
Ritalinic acid^a^	49.33	Highly soluble	−9.35	0.24	High	Yes	No	No	Yes	1.03	0.55
Senkirkine^a^	93.14	Soluble	−7.52	−3.00	High	No	No	No	Yes	1.33	0.55
Renardine^a^	93.14	Soluble	−7.52	−3.00	High	No	No	No	Yes	1.47	0.55
Isonipecotic acid, N‐(3‐methylbutyryl)‐, dodecyl ester^a^	46.61	Moderately soluble	−3.71	−5.51	High	Yes	No	Yes	Yes	5.54	0.55
Benzoic acid^b^	37.30	Soluble	−5.72	−2.20	High	Yes	No	No	Yes	1.44	0.85
Carbonic acid (monoamide, N‐pentyl‐, hexyl ester)^b^	38.33	Soluble	−4.72	−3.01	High	Yes	No	No	Yes	3.36	0.55
Benzoquinoline^b^	12.89	Soluble	−4.98	−3.83	High	Yes	Yes	Yes	Yes	3.13	0.55
4‐Dodecylmorpholine^b^	12.47	Moderately soluble	−3.97	4.14	High	Yes	No	Yes	Yes	4.31	0.55
Madecassoside^b^	335.44	Moderately soluble	−13.13	−4.44	Low	No	Yes	No	No	−1.36	0.17
Furostane^b^	26.30	Poorly soluble	−3.16	−7.02	Low	No	No	No	Yes	6.05	0.55
Benzamide^b^	43.09	Very soluble	−6.58	−1.42	High	Yes	No	No	Yes	0.94	0.55
Sarcosine^b^	49.33	Highly soluble	−8.82	1.49	High	No	No	No	Yes	−1.34	0.55
Oleic diethanolamide^b^	60.77	Moderately soluble	−4.15	−4.72	High	Yes	No	No	Yes	4.97	0.55
Bicuculline^b^	66.46	Moderately soluble	−6.69	−4.02	High	Yes	No	Yes	Yes	2.48	0.55
Oxoglaucine^b^	66.88	Moderately soluble	−5.99	−4.39	High	Yes	No	Yes	Yes	2.99	0.55
Nicotinate mononucleotide^b^	167.44	Very soluble	−10.30	−0.06	Low	No	No	No	Yes	−2.84	0.11
Kynurenic acid^b^	70.16	Soluble	−6.54	−2.29	High	No	No	No	Yes	1.20	0.85
N‐(3‐chlorophenyl)‐3‐methoxybenzamide^b^	38.33	Soluble	−5.75	−3.59	High	Yes	No	Yes	Yes	3.10	0.55
2‐Cyclohexylamino‐5‐nitropyridine^b^	70.74	Soluble	−5.65	−3.06	High	Yes	No	Yes	Yes	1.67	0.55
Pantothenic acid^b^	106.86	Very soluble	−8.40	−0.06	High	No	No	No	Yes	−0.48	0.56
Xanthurenic acid^b^	90.39	Soluble	−6.88	−2.13	High	No	No	No	Yes	0.81	0.56
Indole‐3‐pyruvate^b^	70.16	Soluble	−6.62	−2.16	High	Yes	No	No	Yes	1.14	0.85

^a^ *Phoenix dactylifera* L.

^b^ *Phoenix dactylifera*.

### 10.5 Molecular Docking Analysis

The lead compounds showed lower docking energies along with strong hydrogen bonding and favorable hydrophobic/π–alkyl interactions with key catalytic residues, indicating stable and high‐affinity binding within the enzyme active sites. These interactions increase ligand specificity and stabilize the ligand–protein complex, thereby improving the likelihood of effective enzyme inhibition. Therefore, binding energy and interaction are considered key indicators of pharmacological potential as they collectively reflect its binding strength, stability, and probable biological efficacy. The docking study revealed that the selected compounds exhibited strong interactions with key residues of tyrosinase, elastase, and hyaluronidase enzymes, along with notable binding affinities (Table 6). For tyrosinase, 6‐sec‐butylquinoline interacted with LEU A: 239, PRO A: 225, and MET A: 253, showing a docking score of −9.87 kcal/mol, while flavone formed interactions with LEU A: 239, PHE A: 252, and LYS A: 140, with a score of −9.75 kcal/mol. Myrtenol bound to LYS A: 249, PHE A: 217, and LEU A: 218, exhibiting a docking score of −8.838 kcal/mol. Similarly, 2‐(methylthio)benzothiazole interacted with TYR B: 374, LEU B: 344, and ALA B: 346 with a docking score of −9.165 kcal/mol. The compound isonipecotic acid, N‐(3‐methylbutyryl)‐, dodecyl ester showed the strongest binding among tyrosinase complexes, interacting with PHE A: 46, GLY A: 302, and TYR A: 301 and achieving a docking score of −15.82 kcal/mol. Benzoic acid interacted with LYS B: 409, PHE B: 377, and LYS B: 300, with a docking score of −8.60054 kcal/mol, while carbonic acid (monoamide, N‐pentyl‐, hexyl ester) showed weaker binding with TYR A: 121, ASN A: 153, and LYS A: 151 and a score of −6.55734 kcal/mol. Benzoquinoline interacted with ASN A: 215, HIS A: 222, and PHE A: 241, with a docking score of −10.0097 kcal/mol. In the case of elastase, 6‐sec‐butylquinoline interacted with VAL A: 235, LEU A: 214, and PHE A: 248, showing a docking score of −10.52 kcal/mol, while flavone bound to VAL A: 140, LEU A: 250, and PHE A: 213, with a score of −10.39 kcal/mol. Myrtenol interacted with TYR A: 132, ALA A: 133, and LYS A: 136, yielding a docking score of −9.21 kcal/mol. 2‐(methylthio)benzothiazole formed interactions with TRP A: 203, LEU A: 181, and THR A: 215, with a docking score of −8.11 kcal/mol. Isonipecotic acid, N‐(3‐methylbutyryl)‐, dodecyl ester again showed strong binding with TYR A: 132, LYS A: 136, and PHE A: 213, with a docking score of −13.01 kcal/mol. Benzoic acid interacted with GLU A: 219, VAL A: 217, and PHE A: 138, with a docking score of −8.83959 kcal/mol, while carbonic acid (monoamide, N‐pentyl‐, hexyl ester) showed weaker interaction with TYR A: 121, ASN A: 153, and ARG A: 117 and a docking score of −6.7697 kcal/mol. Benzoquinoline interacted with ALA A: 133, LEU A: 212, and THR A: 304, with a docking score of −10.545 kcal/mol. For hyaluronidase, 6‐sec‐butylquinoline interacted with TRP A: 184, TYR A: 55, and TYR A: 227, showing a docking score of −12.44 kcal/mol, while flavone bound to TYR A: 227, VAL A: 109, and ILE A: 53, with a score of −12.12 kcal/mol. Myrtenol interacted with TYR A: 55, PRO A: 18, and GLY A: 302, with a docking score of −9.20 kcal/mol. In a study, the dewandaru seed extract demonstrated strong anti‐elastase activity with IC_50_ value of 114 μg/mL. A lower IC_50_ value indicates greater inhibitory activity. Kinetic measurements of porcine pancreatic elastase (PPE) activity were determined using Suc‐(Ala)3‐p‐nitroanilide as a substrate and polyphenolic compounds as inhibitors (Theansungnoen et al. 2022). They can bind to enzymes through hydrogen interactions between the hydroxyl groups and their amino acid side chains to break down and inhibit the catalytic activity of enzymes. High anti‐elastase activity has been documented due to the presence of phenolic and flavonoids (Kosasih et al. 2025). Furthermore, the presence of phenolic substances (carotenoids, flavonoids, and polyphenols) is often responsible for plants' powerful antioxidant action, also another character responsible for anti‐aging capability. A phytochemical analysis of the *E. uniflora* seed extract revealed the presence of key compounds, including ellagic acid, quercetin, and kaempferol. Several compounds of *E. uniflora* seed extract that are associated with elastase inhibition activity are gallic acid, myricitrin, and myricetin were phenolic acids in nature (Wenas et al. 2024). Gallic acid is known for its ability to suppress inflammation and oxidative stress. Polyphenols like quercetin, myricitrin, and myricetin are able to form hydrogen bonds and bind to amino acid groups of the elastase. These will eliminate the catalytic activity site and cause the denaturation of the elastase enzyme (Lin et al. 2025). The binding process provokes a hydrophobic effect that produces complexes that are not soluble in water; then, elastase will be denatured. Research shows that the seed extract may prevent premature aging of the skin induced by UV with the ability to increase the elasticity of the skin. The elastase inhibitory activities of 2‐(3‐phenylpropyl) phenol and 5‐phenylvaleric acid observed in a study showed promising alignment with prior findings on effective elastase inhibitors. Both compounds demonstrated strong binding affinity toward key catalytic residues—HIS57, SER195, and VAL216—which are essential for the proteolytic activity of elastase (Wenas et al. 2025). Their molecular frameworks, which include hydrophobic alkyl chains and aromatic rings, contribute significantly to interaction stability through Pi–alkyl and hydrophobic contacts, in line with earlier structure–activity relationship (SAR) studies on elastase inhibitors (Aati et al. 2023). The phenolic group compound also provides additional hydrogen bonding potential, which has been suggested to improve binding specificity (Ramadan 2023). 5‐phenylvaleric acid, with its extended aliphatic chain and terminal carboxyl group, mimics features of fatty‐acid‐derived inhibitors, which often leverage the flexibility of their side chains to achieve optimal alignment within the S1 pocket of elastase (Su et al. 2024).

**TABLE 6:** Molecular docking of *Phoenix dactylifera L.* and *Phoenix dactylifera* derived phytochemicals against tyrosinase, hyaluronidase, and elastase.

Compounds	Interacting residues	Docking score	2D conformation
6‐sec‐Butylquinoline/Tyrosinase^a^	LEU A: 239 PRO A: 225 MET A: 253	−9.87 kcal/mol	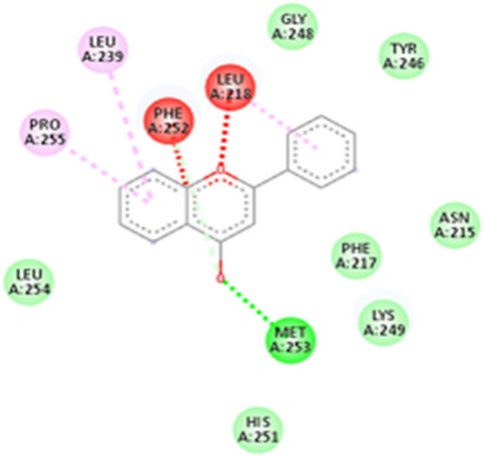
Flavone/Tyrosinase^a^	LEU A: 239 PHE A: 252 LYS A: 140	−9.75 kcal/mol	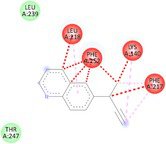
Myrtenol/Tyrosinase^a^	LYS A: 249 PHE A: 217 LEU A: 218	−8.838 kcal/mol	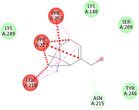
2‐(Methylthio)benzothiazole/Tyrosinase^a^	TYR B: 374 LEU B: 344 ALA B: 346	−9.165 kcal/mol	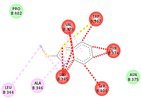
Isonipecotic acid, N‐(3‐methylbutyryl)‐, dodecyl ester/Tyrosinase^a^	PHE A: 46 GLY A: 302 TYR A: 301	−15.82 kcal/mol	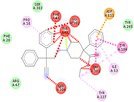
Benzoic acid/Tyrosinase^b^	LYSB: 409 PHE B: 377 LYS B: 300	−8.60054 kcal/mol	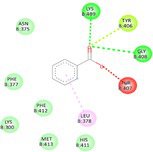
Carbonic acid (monoamide, N‐pentyl‐, hexyl ester)/Tyrosinase^b^	TYR A: 121 ASN A: 153 LYS A: 151	−6.55734 kcal/mol	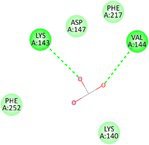
Benzoquinoline/Tyrosinase^b^	ASN A: 215 HIS A: 222 PHE A: 241	−10.0097 kcal/mol	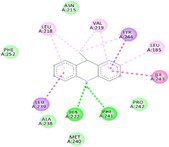
6‐sec‐Butylquinoline/Elastase^a^	VAL A: 235 LEU A: 214 PHE A: 248	−10.52 kcal/mol	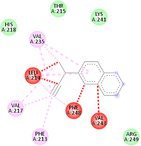
Flavone/Elastase^a^	VAL A: 140 LEU A: 250 PHE A: 213	−10.39 kcal/mol	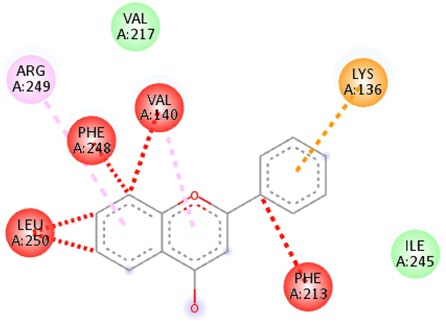
Myrtenol/Elastase^a^	TYR A: 132 ALA A: 133 LYS A: 136	−9.21 kcal/mol	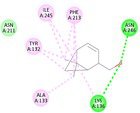
2‐(Methylthio)benzothiazole/Elastase^a^	TRP A: 203 LEU A: 181 THR A: 215	−8.11 kcal/mol	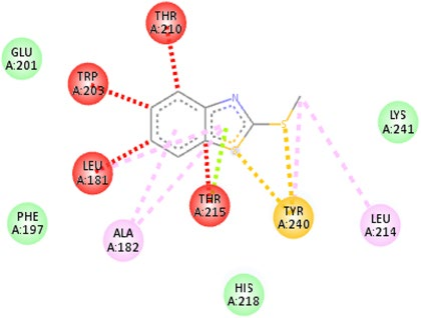
Isonipecotic acid, N‐(3‐methylbutyryl)‐, dodecyl ester/Elastase^a^	TYR A: 132 LYS A: 136 PHE A: 213	−13.01 kcal/mol	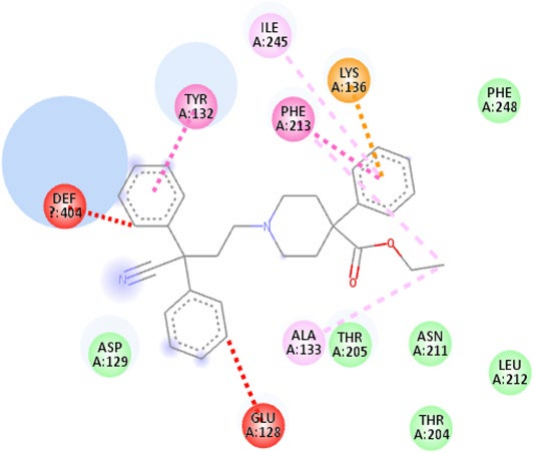
Benzoic acid/Elastase^b^	GLU A: 219 VAL A: 217 PHE A: 138	−8.83959 kcal/mol	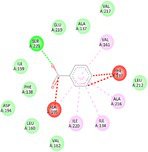
Carbonic acid (monoamide, N‐pentyl‐, hexyl ester)/Elastase^b^	TYR A: 121 ASN A: 153 ARG A: 117	−6.7697 kcal/mol	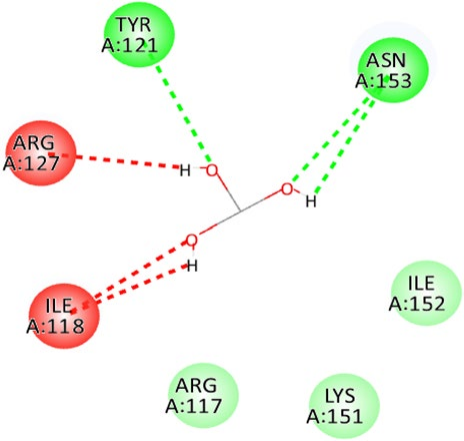
Benzoquinoline/Elastase^b^	ALA A: 133 LEU A: 212 THR A: 304	−10.545 kcal/mol	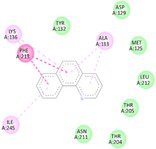
6‐sec‐Butylquinoline/Hyaluronidase^a^	TRP A: 184 TYR A: 55 TYR A: 227	−12.44 kcal/mol	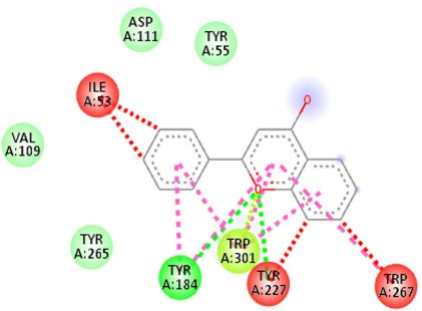
Flavone/Hyaluronidase^a^	TYR A: 227 VAL A: 109 ILE A: 53	−12.12 kcal/mol	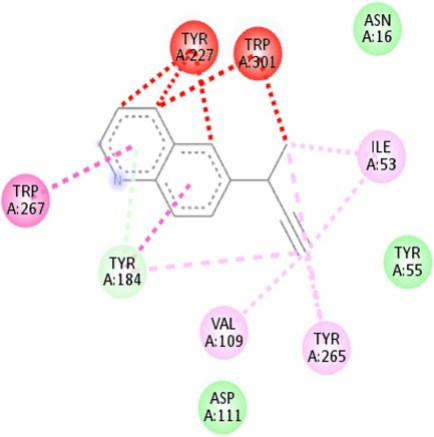
Myrtenol/Hyaluronidase^a^	TYR A: 55 PRO A: 18 GLY A: 302	−9.20 kcal/mol	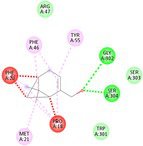
2‐(Methylthio)benzothiazole/Hyaluronidase^a^	TYR A: 227 ILE A: 53 ASP A: 111	−10.31 kcal/mol	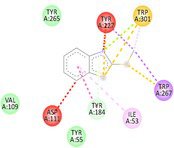
Isonipecotic acid, N‐(3‐methylbutyryl)‐, dodecyl ester/Hyaluronidase^a^	GLY A: 302 TYR A: 55 TRP A: 301	−15.49 kcal/mol	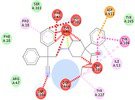
Benzoic acid/Hyaluronidase^b^	GLN A: 121 PRO A: 117 MET A: 212	−8.51412 kcal/mol	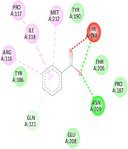
Carbonic acid (monoamide, N‐pentyl‐, hexyl ester)/Hyaluronidase^b^	LEU A: 283 ARG A: 330 PRO A: 326	−6.6509 kcal/mol	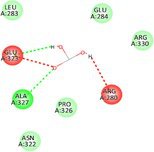
Benzoquinoline/Hyaluronidase^b^	TRP A: 301 TYP A: 265 VAL A: 109	−10.4517 kcal/mol	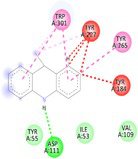

^a^ *Phoenix dactylifera* L.

^b^ *Phoenix dactylifera*.

## 11 Conclusion

This study demonstrates that Zahidi dates ( *Phoenix dactylifera* ) possess superior anti‐aging potential compared to *Phoenix dactylifera* L. (Piarom dates), which may be attributed to their higher phenolic and flavonoid contents along with stronger free radical scavenging capacity. The elevated levels of antioxidant enzymes such as superoxide dismutase (SOD), catalase, and peroxidase further reinforce their antioxidant effectiveness. Moreover, both extracts exhibited significant inhibitory activity against extracellular matrix (ECM)‐degrading enzymes, including elastase (49.69% ± 1.15%), tyrosinase (58.41% ± 1.98%), and hyaluronidase (51.65% ± 1.49%), indicating their potential in mitigating skin aging processes. In silico results also confirmed that the main phytochemicals possess favorable skin permeability and gastrointestinal absorption profiles, supporting their suitability for dermal applications. Molecular docking analysis identified 6‐sec‐butylquinoline from *Phoenix dactylifera* and isonipecotic acid from *Phoenix dactylifera* L. as the most promising lead compounds based on their high binding affinities. Overall, these findings provide valuable evidence for the development of novel cosmeceutical or pharmaceutical products derived from natural or semi‐synthetic sources; however, the study is limited by its reliance on in vitro assays and computational predictions, which may not fully replicate physiological conditions. Additionally, the absence of in vivo and clinical validation, along with the lack of assessment of long‐term safety, bioavailability, and synergistic effects within whole extracts, restricts direct clinical translation, thereby necessitating further experimental and clinical investigations to confirm efficacy and safety.

## Author Contributions

**Farheen Asaad:** investigation, data curation, methodology, software, writing – original draft. **Mazhar Abbas:** conceptualization, project administration, supervision, resources, validation. **Waqas Haider:** investigation, methodology, formal analysis, writing – original draft. **Naveed Munir:** validation, visualization, writing – original draft. **Maha Gul Zafar:** investigation, methodology, formal analysis, writing – review and editing. **Muhammad Riaz:** validation, visualization, writing – review and editing. **Muhammad Arshad:** validation, visualization, formal analysis, writing – review and editing. **Muhammad Arfan Zaman:** formal analysis, validation, visualization, writing – review and editing. **Tariq Hussain:** validation, visualization, writing – review and editing, formal analysis. **Muhammad Adnan Ayub:** validation, visualization, writing – review and editing, formal analysis. **Hasan Ejaz:** formal analysis, visualization, writing – review and editing. **Quzi Sharmin Akter:** formal analysis, visualization, writing – review and editing.

## Funding

The authors have nothing to report.

## Disclosure

All authors have read and approved the final version of the manuscript. Mazhar Abbas and Quzi Sharmin Akter had full access to all of the data in this study and take complete responsibility for the integrity of the data and the accuracy of the data analysis.

## Ethics Statement

This study did not involve any human or animal subject as this is an in vitro study. However, the study plan was approved by the research committee of the College of Veterinary and Animal Sciences, Jhang, Pakistan.

## Conflicts of Interest

The authors declare no conflicts of interest.

## Data Availability

Data will be available from the principal and corresponding authors on reasonable request.
